# Architecture of a Smart Home for Aging in Place

**DOI:** 10.1093/geroni/igaa057.126

**Published:** 2020-12-16

**Authors:** Neda Norouzi

**Affiliations:** University of Texas at San Antonio, San Antonio, Texas, United States

## Abstract

The United States Department of Health and Human Services (2017) estimates that there are 65 million people age 60+ residing across the fifty states. A national survey conducted by the American Association of Retired Persons (AARP) indicates that 76% of people ages 55+ prefer to age-in-place and live independently (2018). The Census Bureau American Community Survey (2015) estimates that 13 million adults have difficulties living independently, 80% of which receive assistance in their private homes. However, only 50% of these homes meet the physical needs of people who choose to age-in-place (AARP, 2018). Recent advancements in technology have led to the development of smart homes. Technology can support aging-in-place and independent living by offering necessary tools for building systems that identify behavioral patterns and offer automated decision-making. However, not all older adults are customed to using technology or comfortable with being monitored with artificial intelligence (Wang et al., 2019). In response to this concern, the current study used grounded theory framework to analyze 62 interviews of people ages 55-93 to indicate if and how older adults prefer to utilize technology in their homes. The results of the study presented that while some older adults felt they might be too old to learn and use technology, nearly 85% of the interviewers agreed that incorporating technology in the built environment could benefit them. They are especially willing to learn and use technology in their homes when the benefits are related to their health, social and emotional connection, entertainment, safety, and daily chores.

